# A Differential Diagnosis of Merc. Iod, Ruber and Merc. Iod. Flav., in Diphtheria

**Published:** 1881-03

**Authors:** C. Carleton Smith

**Affiliations:** Phila.


### DIFFERENTIAL DIAGNOSIS OF MERC. IOD. FLAV. AND MERC. IOD. RUBER, IN DIPHTHERIA.

BY C. CARLETON SMITH, M.D., PHILA.

| *Flavus.* | *Ruber.* |
|---|---|
| 1. Both have tonsils, uvula and pharynx swollen. *Slightly* swollen in flavus. | 1. Tonsils *greatly* swollen and very painful, with prominent enlargement of sub maxillary glands. |
| 2. Disposition to hawk, which is caused by excessive secretion of mucus, very *difficult* to dislodge. | 2. Same disposition to hawk, but here is caused by a sensation as of a lump in throat; occasionally this so-called lump is hawked up, when it is found to consist of hardened green mucus. |
| 3. Posteria wall of pharynx is found studded all over with *spots* looking like small *ulcerated* points. | 3. These not found under the bin-iod. |
| 4. Severe burning in right side of *neck* as if coal of fire was laying on it, at the same | 4. Burning is *in* throat and feels as if *recently scalded*, and with this we have outside |

| | |
|---|---|
| time stiffness of neck. (*Phytolac.* red-hot ball in fauces). | *glandular swellings*, sore to touch. But no *stiffness* of neck. Patches of inflammation in larynx which become livid purple. |
| 5. Throat and nose *dry* as if closed up *with mucus.* | 5. Throat and nose dry, but without the feeling of being closed up or obstructed. |
| 6. Disposition to swallow frequently, from a feeling of swelling in throat. | 6. Constant disposition to swallow on account of a large collection of water in mouth. |
| 7. Has profuse secretion of mucus in throat, the effort to dislodge which causes retching. (Patients vomit, not from nausea, but from trying to raise this mucus.) | |
| 8. Both drugs have enlarged glands. | 8. Throat looks much *less red* and *angry.* |
| 9. One of the most important indications for use of *Merc. Iod. Fl.* is the condition of the tongue. The back portion only of tongue is coated a *bright yellow*, becoming somewhat darker if it remains long; through this coating can be seen the *enlarged* papillæ, quite red, standing up; while anterior portion of tongue is *clean. Mouth* is *dry*, but not the tongue. | 9. Great dryness of tongue with desire to wet the mouth, also feeling as if tongue and whole buccal cavity were badly scalded with some hot fluid. |
| 10. Lips *dry* and *burning*, especially the lower. | 10. Lips very slimy, so that they *stick together.* |

CONCOMITANTS.

| | |
|---|---|
| 11. Mental, a decided tend ency to *destroy* on part of pa tient. | 11. Patient low spirited and weeps very easily. |
| 12. Taste, craves acid *drink*. | 12. Wants food well *salted*, but still he drinks but little. |
| 13. Bright red fine eruption on *chest* and *abdomen*. | 13. Syphilitic ulcers over skin. |

*Mercurius*, *vivus*, *corr.*, *jod.*, *sulphate* and *cinnab.*, all have one symptom in common, viz., pain through *right* chest to back.

